# Ozone ultrafine bubble water inhibits the early formation of *Candida albicans* biofilms

**DOI:** 10.1371/journal.pone.0261180

**Published:** 2021-12-10

**Authors:** Yuka Shichiri-Negoro, Chiaki Tsutsumi-Arai, Yuki Arai, Kazuhito Satomura, Shinichi Arakawa, Noriyuki Wakabayashi

**Affiliations:** 1 Department of Removable Partial Prosthodontics, Graduate School, Tokyo Medical and Dental University (TMDU), Tokyo, Japan; 2 Department of Oral Medicine and Stomatology, Tsurumi University School of Dental Medicine, Yokohama, Kanagawa, Japan; 3 Department of Lifetime Oral Health Care Science, Graduate School of Medical and Dental Sciences, Tokyo Medical and Dental University (TMDU), Tokyo, Japan; University of the Witwatersrand, SOUTH AFRICA

## Abstract

This study aimed to investigate the effect of ozone ultrafine bubble water (OUFBW) on the formation and growth of *Candida albicans* (*C*. *albicans*) biofilms and surface properties of denture base resins. OUFBWs were prepared under concentrations of 6 (OUFBW6), 9 (OUFBW9), and 11 ppm (OUFBW11). Phosphate buffered saline and ozone-free electrolyte aqueous solutions (OFEAS) were used as controls. Acrylic resin discs were made according to manufacturer instructions, and *C*. *albicans* was initially cultured on the discs for 1.5 h. A colony forming unit (CFU) assay was performed by soaking the discs in OUFBW for 5 min after forming a 24-h *C*. *albicans* biofilm. The discs after initial attachment for 1.5 h were immersed in OUFBW and then cultured for 0, 3, and 5 h. CFUs were subsequently evaluated at each time point. Moreover, a viability assay, scanning electron microscopy (SEM), Alamar Blue assay, and quantitative real-time polymerase chain reaction (qRT-PCR) test were performed. To investigate the long-term effects of OUFBW on acrylic resin surface properties, Vickers hardness (VH) and surface roughness (Ra) were measured. We found that OUFBW9 and OUFBW11 significantly degraded the formed 24-h biofilm. The time point CFU assay showed that *C*. *albicans* biofilm formation was significantly inhibited due to OUFBW11 exposure. Interestingly, fluorescence microscopy revealed that almost living cells were observed in all groups. In SEM images, the OUFBW group had lesser number of fungi and the amount of non-three-dimensional biofilm than the control group. In the Alamar Blue assay, OUFBW11 was found to suppress *Candida* metabolic function. The qRT-PCR test showed that OUFBW down-regulated *ALS1* and *ALS3* expression regarding cell-cell, cell-material adhesion, and biofilm formation. Additionally, VH and Ra were not significantly different between the two groups. Overall, our data suggest that OUFBW suppressed *C*. *albicans* growth and biofilm formation on polymethyl methacrylate without impairing surface properties.

## Introduction

Oral candidiasis is one of the most common oral infections in patients receiving immunotherapy and in older individuals with weak immunity [1]. Oral candidiasis related to wearing dentures is called denture stomatitis (DS) [2, 3]. *Candida albicans* (*C*. *albicans*), a pathogenic fungus abundant in denture plaque, is highly associated with DS [4, 5]. *C*. *albicans* on the denture surface adheres to other bacteria and acts as a scaffold for biofilms [6]. Consequently, the adherence and subsequent increase of *C*. *albicans* on the denture surface accelerate biofilm formation [7]. Additionally, biofilms have a stronger drug resistance than free-floating microorganisms [8]. Therefore, preventing the adherence and increase of *C*. *albicans* on dentures and suppressing denture biofilm formation early is the first step in preventing DS.

Various therapeutic strategies have been developed for DS, but none have been clearly established. Oral antifungal agents, such as amphotericin B, nystatin, and miconazole, as well as systemic antifungal drugs, have been used for DS treatment [9]. However, the evidence on their effects is limited, and antifungal agents may confer resistance [10]. Moreover, brushing, preservatives, and disinfectants have occasionally been used to remove plaque on denture surfaces [11, 12]. However, these methods cannot completely remove *C*. *albicans* and may immediately increase the concentration of the remaining *C*. *albicans* on denture base resin.

Ozone ultrafine bubble water (OUFBW) contains very small ozone particles (< 200 nm in diameter) to overcome unstable ozonated water [13], which has a half-life of about only 20 min and will degrade back into oxygen. Owing to microbubble characteristics, the gas-water interface of nanobubbles contains OH^-^ ions distributed on H^+^ ions and functions as a shell to prevent gas dispersion [14]. The ozone in OUFBW remains stable for more than 6 months and protects against exposure to ultraviolet light. In mucosal disease models, OUFBW induces an oxidative stress response and consequently enhances healing [15]. OUFBW can suppress periodontal pathogens and clinically improve periodontal status [16]. Additionally, OUFBW has been reported to induce cellular reactions that generate reactive oxygen species in regenerative periodontal tissue [17]. In contrast, ozone can suppress the pathogenicity and hyphal growth of *C*. *albicans* [18], indicating that OUFBW may inhibit the progression of *C*. *albicans* on denture base resin. Before implementing a clinical trial, the potential antifungal effects of OUFBW on denture base resin and the ozone appropriate concentration should be clarified through an in vitro study. Accordingly, this study aimed to investigate the influence of OUFBW on *C*. *albicans* adherence and early biofilm formation in denture base resin.

## Materials and methods

### Preparation of OUFBW solutions

OUFBW was supplied by Nippon Beatty Lease Co., Ltd. Nanosui Company (Tokyo, Japan). It requires electrolytes, including sodium, calcium, magnesium, and potassium to stabilize nanobubbles [19]. Accordingly, OUFBWs were prepared by adding 6 (OUFBW6), 9 (OUFBW9), and 11 (OUFBW11) ppm ozone ultrafine bubbles to water containing electrolytes. The ozone concentration of each solution was measured using an ozone meter (AOM-05, Sato Shoji Co., Ltd., Japan) and immediately used for subsequent investigations. Ozone-free electrolyte aqueous solution of OUFBW11, which had the highest ozone concentration among all OUFBWs, was referred to as OFEAS since the electrolyte concentration increases proportionally with the ozone concentration [19]. OFEAS and phosphate buffered saline (PBS; pH 7.2) were used as controls.

### Sample preparation

A total of 119 square-shaped specimens (10 × 10 × 2 mm^3^) were prepared from polymethyl methacrylate (PMMA) denture base resin (ACRON, GC, Tokyo, Japan) by sectioning three cuboid PMMA samples (10 × 10 × 80 mm^3^) into disks [20]. The upper and lower surfaces of each denture base resin disc was polished using a 320-grit abrasive paper under dry conditions. The surface roughness (Ra) of each specimen was determined by a profilometer (Surfcom Flex, Seimitsu, Tokyo, Japan), and the mean value of two measurements was 1.24 ± 0.15 μm. All denture base resin specimens used in the *Candida* experiment were sterilized using ethylene oxide gas (EOG), stored in a sterilization chamber at 40°C for 24 h to remove residual EOG, and immediately used for testing.

### *Candida* growth conditions

Cryopreserved *C*. *albicans* specimens (ATCC 18804) were seeded on a Sabouraud glucose agar plate (Kanto Chemical Co., Inc., Tokyo) until *C*. *albicans* colonies formed. Colonies were picked using an inoculation loop, seeded into Tryptic soy broth supplemented with 5% dextrose (TSBD; Becton, Dickinson and Company, New Jersey, USA), and aerobically cultured on a shaker at 75 rpm and 30°C for 5 h same as the previous reports [21, 22]. Yeast cells in the mid-log phase were standardized at 10^6^ cells/mL in a TSBD medium using a OneCell Counter (Bio Chemical Science, Tokyo, Japan).

### Cell adhesion on denture base materials

Each specimen was placed in one of the wells of a 24-well plate comprising 500 μL of artificial saliva containing 1.25 mM of Ca(NO_3_)_2_4H_2_O, 0.90 mM of KH_2_PO_4_, 129.91 of mM KCl, 59.93 of mM Tris buffer, and 2.2 g/L of porcine gastric mucin (pH 7.4) [22]. Plates were incubated for 60 min on a shaker at 37°C and 75 rpm and washed twice with 1 mL of PBS (pH 7.2). A *Candida* cell suspension (1 mL) was added to each well containing a disc, and specimens were aerobically maintained for 1.5 h at 37°C during cell adhesion [23]. Subsequently, each specimen was washed twice with 1 mL of PBS to remove non-adhered cells.

### Colony forming unit assay

The Yeast Nitrogen Base (YNB) medium (1 mL) was added to each well containing an initial cell adhesion disc and aerobically incubated for 24 h at 37°C. After washing the specimens twice with PBS, the discs were immersed in a 24-well plate containing 1 mL of each solution of OUFBW6, 9, 11, OFEAS, and control for 5 min. After cleaning, the discs were washed twice with PBS. Cells were scraped using a cell scraper, and the attached cells were dissociated by pipetting. Fungal suspension was serially diluted and spread on a Sabouraud glucose agar plate. CFUs were counted after aerobically culturing the plates for 24 h at 37°C.

### *Candida* growth assay

To investigate whether OUFBW inhibits *Candida* proliferation, the initial cell adhesion disc was immersed in 1 mL of each solution of OUFBW6, 9, 11, OFEAS, and control for 5 min. After treatment, the specimens were washed twice with PBS and cultured in a YNB medium for 0, 3, and 5 h. The *Candida* cells on the sample were stamped to Sabouraud glucose agar plates [24]. After 12 h of incubation at 37°C, the number of colonies on plates was counted.

### Viability assay

In this assay, the initial cell adhesion discs were immersed in 1 mL of each solution of OUFBW6, 9, 11, OFEAS and control for 5 min, and immediately washed twice with PBS. Each disc was placed in one of the wells of a 24-well plate. Subsequently, the specimens were stained using the LIVE/DEAD^®^ *Fungal* Light™ Yeast Viability kit (Molecular Probes, Oregon, USA). The kit contained solutions of SYTO^®^9 green-fluorescent nucleic acid and propidium iodide (PI) red-fluorescent nucleic acid stains. Five hundred microliters of PBS, 1 μL of SYTO9, and 1 μL of PI were added to the 24-well plate and incubated at 30°C in darkness. The sample was observed using a fluorescent microscope (BZ-X710; Keyence, Osaka, Japan).

### Scanning electron microscopy (SEM)

For validation of ozone on the morphological change of *C*. *albicans* and the biofilm, the initial cell adhesion discs were washed with OUFBW11 and the control solution, and the specimens were cultured in the YNB medium for 0, 3, and 5 h. After washing the discs twice with PBS and fixing them with 2.5% glutaraldehyde at 4°C for 24 h, each disc was dehydrated in graded concentrations of ethanol (i.e., 50%, 60%, 70%, 80%, and 90%; absolute ethanol), transferred to liquid t-butyl alcohol mediums, and stored in a freezer at −20°C until butyl alcohol froze. Subsequently, the sample was transferred to a freeze drying device (ID-2; Eiko Engineering, Tokyo, Japan) to sublimate t-butyl alcohol. The specimens were attached to an aluminum stub and observed using SEM (JCM-6000 NeoScope; JEOL Ltd., Tokyo, Japan). Gold was coated using an ion sputter coater (SC-701AT; Sanyu Denshi, Tokyo, Japan) [25].

### Alamar Blue assay

The Alamar Blue assay uses a redox indicator assay (alamarBlue®; Bio-Rad Laboratories, California, USA) to measure cell metabolism based on the enzymatic reduction of indicator pigments by viable cells [24]. *C*. *albicans* colonies were aerobically cultured in a TSBD medium for 5 h at 30°C. The *Candida* suspension was confirmed to be standardized at 10^8^ cells/mL, and 100 μL of the prepared *Candida* solution was added to 10 mL of OUFBW11. Moreover, a 10 mL PBS solution was used as a control. Following reaction for 5 min, 10 μL of the acquired solution was placed in a 96-well plate with 10 μL of 10% of the redox indicator assay and 90 μL of the TSBD medium. Following incubation at 37°C for 24 h, absorbance was measured using a multidetection reader (LabSystems Multiskan®; MultiSoft, Helsinki, Finland) at a 570-nm wavelength. According to the manufacturer’s instructions, the Alamar Blue values of each sample were compared by absorbance.

### Quantitative real-time polymerase chain reaction (qRT-PCR) analysis

*C*. *albicans* colonies were cultured in the TSBD medium at 75 rpm and 30°C for 5 h. *C*. *albicans* cells were standardized at 10^6^ cells/mL and centrifuged at 4,000 rpm for 10 min to collect fungal cells. The supernatant was discarded, and the precipitate was mixed with 10 mL of the acquired solution. OUFBW11 was used for cleaning, and PBS was used as a control. Thereafter, after centrifuging at 10,000 rpm for 2 min, the recovered fungus was cultured in a YNB medium for 5 h. Total RNA was extracted using the NucleoSpin RNA Kit (Takara Bio Inc, Tokyo, Japan). *Candida* was washed and recovered by centrifugation, and the yeast cell wall was degraded by the Processing Enzyme Solution and Yeast Processing Buffer to extract RNA and stored at -80°C. The quantity and quality of the extracted total RNA was analyzed by determining absorbance (A260/A280) using a spectrophotometer (NanoDrop 2000; Thermo Fisher Scientific, Massachusetts, USA). RNA samples with a 260/280 ratio of 1.9:2.1 were used. The High-Capacity cDNA Reverse Transcription Kit (Thermo Fisher Scientific, Massachusetts, USA) was used for cDNA synthesis, and the prepared cDNA was diluted 10 times before its usage for RT-PCR. The obtained cDNA was amplified by PCR testing, and product specificity was confirmed by sequencing.

Real-time PCR primers are shown in Table 1, and 18-S *rRNA* was used as a house-keeping gene for reference. Five primers were selected to elucidate the mechanisms of OUFBW on *C*. *albicans*. *ALS1* and *ALS3* were selected as the primers related to cell adhesion and biofilm formation, and *RAS1*, *CPH1*, and *EFG1* were selected as the primers related to hyphal growth [26, 27]. The RT-PCR mixture (25 μL) was freshly prepared and comprised 12.5 μL of SYBR green fluorescent dyes, 1 μL of PCR forward primer, 1 μL of PCR reverse primer, 0.5 μL of cDNA, and 10 μL of RNase-free water. RT-PCR was performed using the StepOnePlus Real Time PCR System (Applied Biosystems, California, USA). Cycling conditions consisted of an initial denaturation step at 95°C for 30 s, followed by 40 cycles at 95°C for 3 s and 60°C for 30 s, and a final dissociation step at 95°C for 15 s and 60°C for 60 s, with a heating rate of 0.3°C/s. All data were normalized to the house-keeping gene *18-S rRNA*, which was considered as the internal reference gene. Relative target-gene expression was calculated as a fold change of 2–ΔΔCt, where ΔCt is the Ct target gene−Ct internal reference genes. All experiments were performed on ice.

**Table 1:** Primers used for qRT-PCR.

Primer	Sequence (5’-3’)
RAS1f	CCCAACTATTGAGGATTCTTATCGTAAA
RAS1r	TCTCATGGCCAGATATTCTTCTTG
EFG1f	CCAGGGTGGTGCTGCTAATAG
EFG1r	GGGTGAAGGGTGAACTGAACC
CPH1f	AACCCGGCATTAGCAGTAGATG
CPH1r	CAGAGTGCTAATGGTGGAGAAGAA
ALS1f	CCCAACTTGGAA TGCTGTTT
ALS1r	TTTCAAAGCGTCGTTCACAG
ALS3f	AATGGTCCTTATGAATCACCATCTACT
ALS3r	GAGTTTTCATCCATACTTGATTTCACA
18srRNAf	CACGACGGAGTTTCACAAGA
18srRNAr	CGATGGAAGTTTGAGGCAAT

### Hardness and surface roughness

Discs were immersed in each solution for 7 days (five discs for each group). Solutions were freshly prepared and changed daily. If the denture at the time of mouthwash reacted to this solution for 1 min daily, the total immersion time for 7 days was equal to approximately 5.5 years. After immersion, all specimens were stored at room temperature. One week after, five specimens from each group were measured using a profilometer (Ra) (Surfcom Flex; Tokyo Seimitsu, Tokyo, Japan) and Vickers hardness (VH) test, which comprised a diamond hardness indenter (AVK-AII; Akashi Seisakusho, Tokyo, Japan) under a load of 300 g for 15 s. Each sample was calculated at two different time points, and the means of both measurements for individual specimens were calculated.

### Statistical analyses

CFU and proliferation assay data were analyzed using the Kruskal-Wallis test, and significant differences between experimental groups were confirmed using the Mann–Whitney U-test and Bonferroni correction test. qRT-PCR results were evaluated by a one-way ANOVA test. The Ra and VH results of each specimen were analyzed using a one-way analysis of variance, followed by Tukey’s significant difference multiple comparison test. A *p*-value < 0.05 was considered statistically significant. Statistical analyses were performed using SPSS version 24.0 (IBM, NY, USA).

## Results

**Fig 1:**
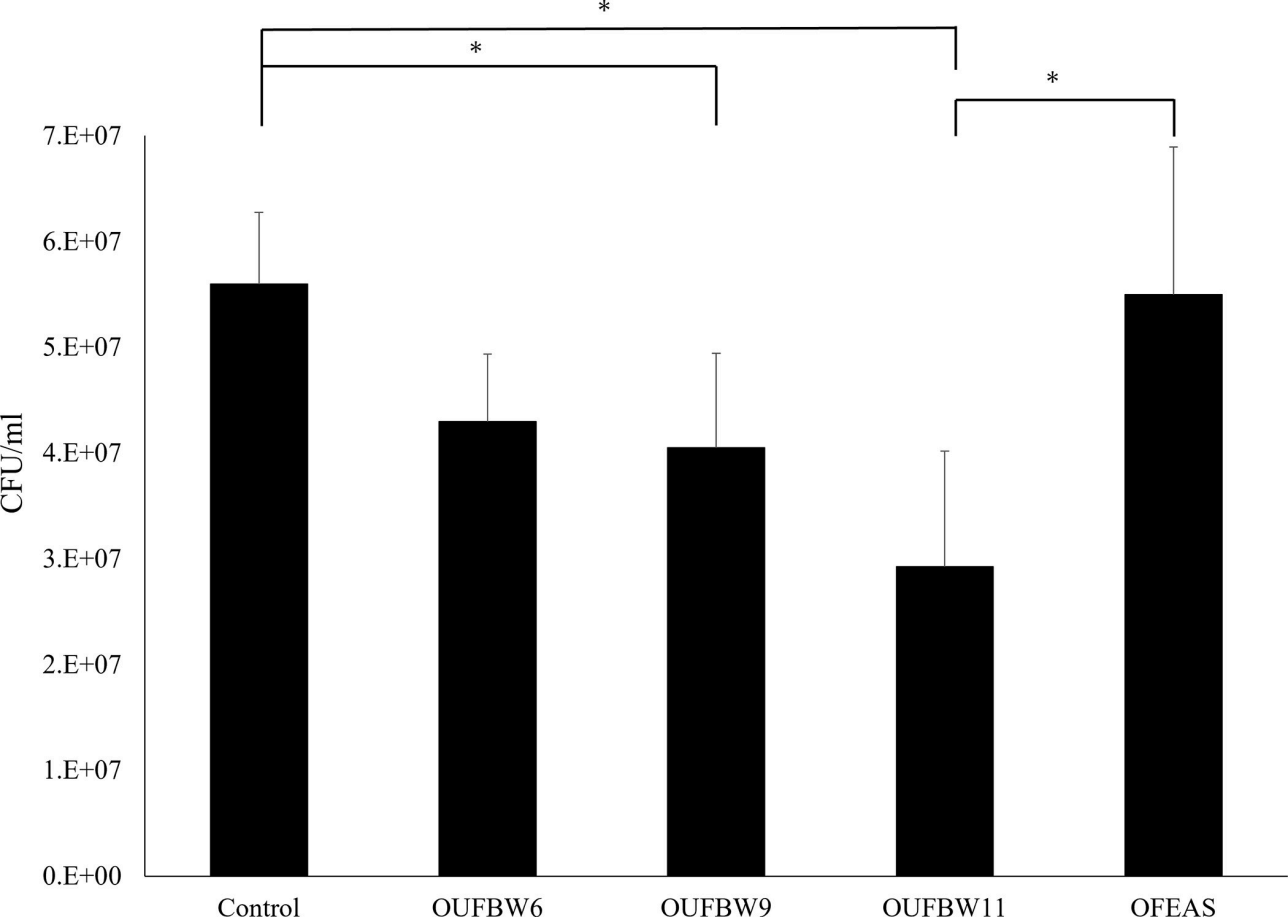
24-h Biofilm quantification of *C*. *albicans* using the CFU assay. Black bars represent the mean colony count detected from specimens with *C*. *albicans* (n = 10 in each group), and the asterisk (*) indicates significant between-group differences (*p* < 0.05).

CFU assay results showed decreased biofilm quantities on the disc surface in association with increased ozone concentrations. The number of CFUs was significantly lower in the OUFBW9 and OUFBW11 groups than in the control group (Fig 1). There were no significant differences in the number of CFUs between the control and OFEAS groups.

**Fig 2:**
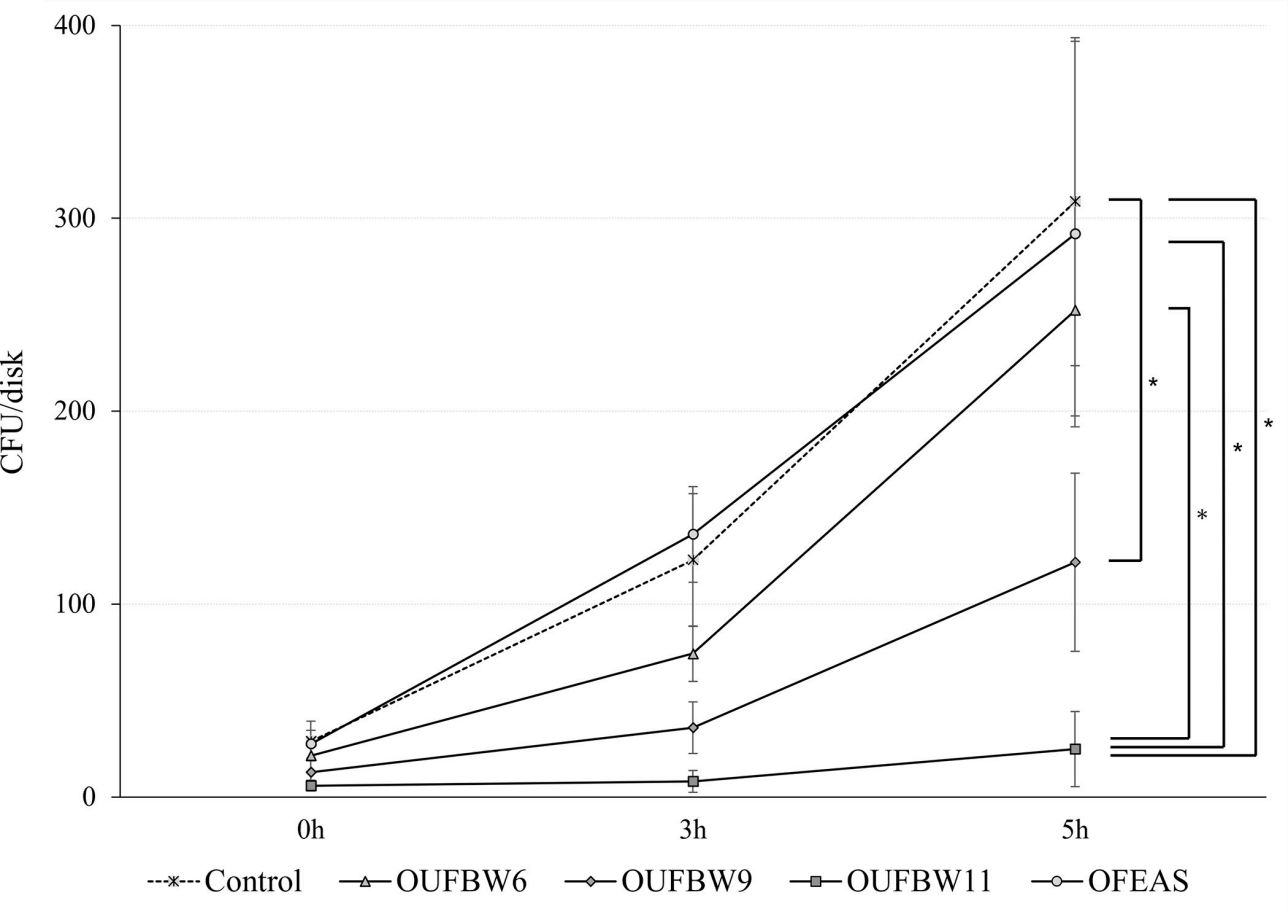
*Candida* growth assay after immersion of samples. After soaking in the cleaning solution, the samples were cultured in a YNB medium for 0, 3, and 5 h and stamped on Sabouraud agar plates. The number of colonies formed on plates were counted (n = 8 at each time point and, in each group). (*: *p* < 0.05).

The *Candida* growth assay indicated that the CFUs increased at each time point in all groups based on culture time. There were no significant differences in the number of colonies between groups directly after immersion. However, the CFU of *C*. *albicans* was significantly lower in the OUFBW11 group than in the control group 3 h following immersion. Moreover, the CFUs were significantly lower in the OUFBW9 and OUFBW11 groups than in the control group 5 h after immersion (Fig 2).

**Fig 3:**
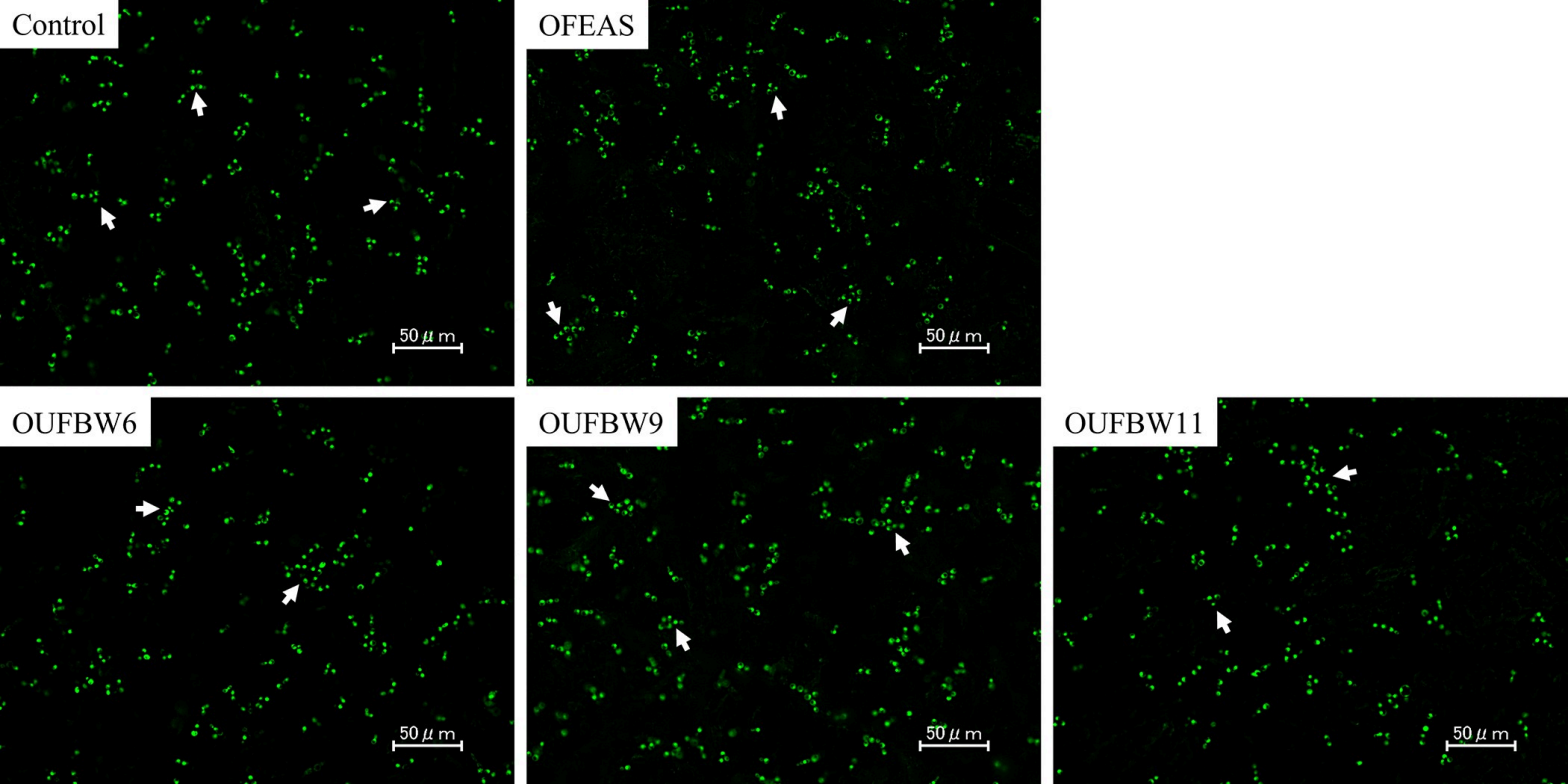
Fluorescence microscope image of *Candida* on PMMA. Green fluorescence indicates live cells, and red fluorescence indicates dead cells. No dead cells were observed in all samples regardless of the increase of ozone concentration.

Fluorescence microscopy, in which green and red fluorescence indicate live cells and dead cells respectively, revealed *C*. *albicans* cell viability in the biofilm. All cells of *C*. *albicans* cells were stained green, and a minimal no number of cells was stained red in all groups (Fig 3).

**Fig 4:**
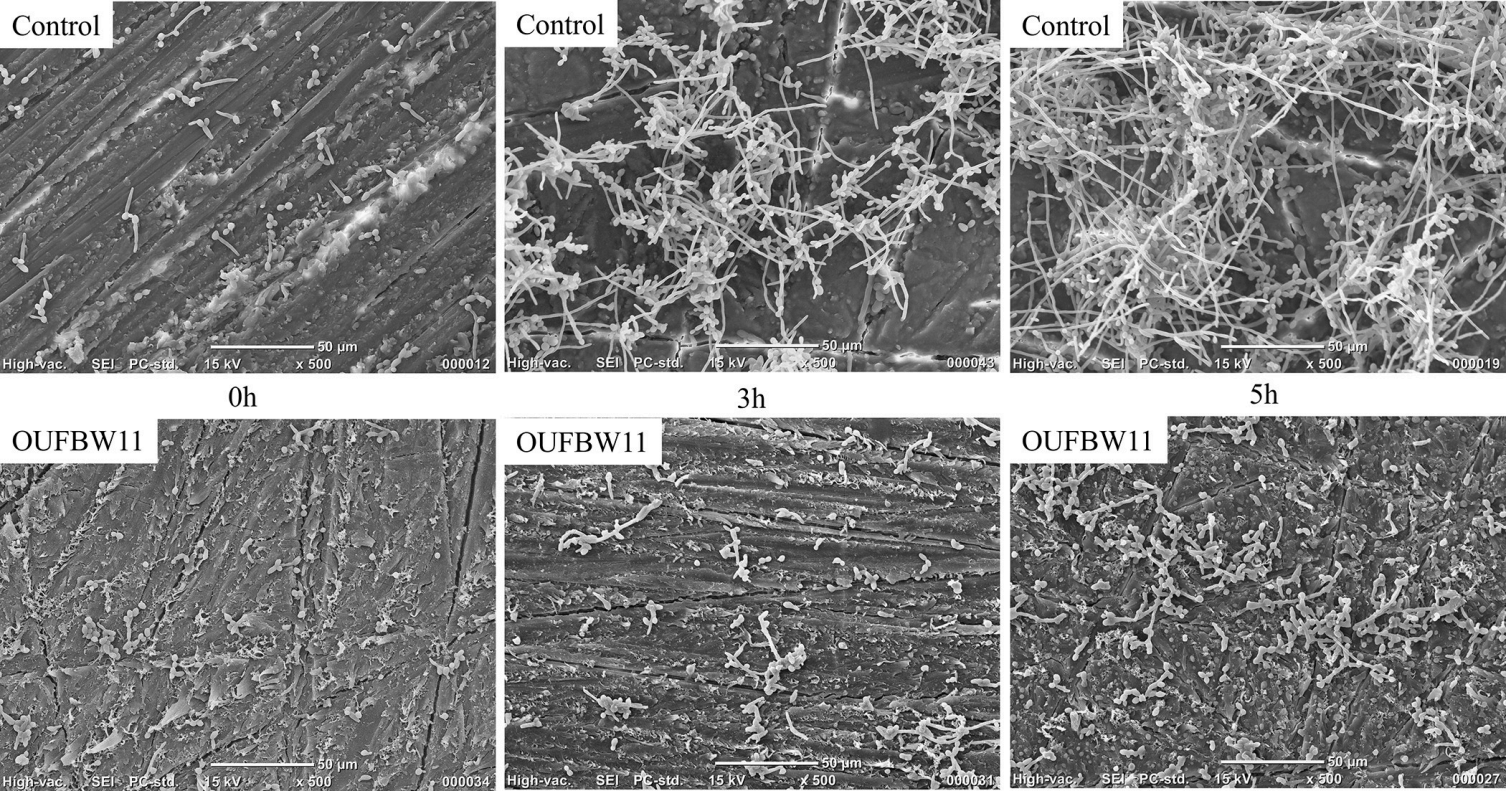
SEM images of *C*. *albicans* biofilms formed on the surface of each group of resin discs. The OUFBW group had fewer fungi and non-three-dimensional biofilms than the control group.

SEM images showed morphological changes in *Candida* biofilm on the denture surface at all time points (0, 3, and 5 h) in both control and OUFBW groups. *C*. *albicans* cells adhered to the denture surface, proliferated, and transformed into a hyphae shape. *C*. *albicans* then increased in number and transformed into three-dimensional morphological biofilms. OUFBW11 treatment inhibited the growth and accumulation of *C*. *albicans* (Fig 4).

**Fig 5:**
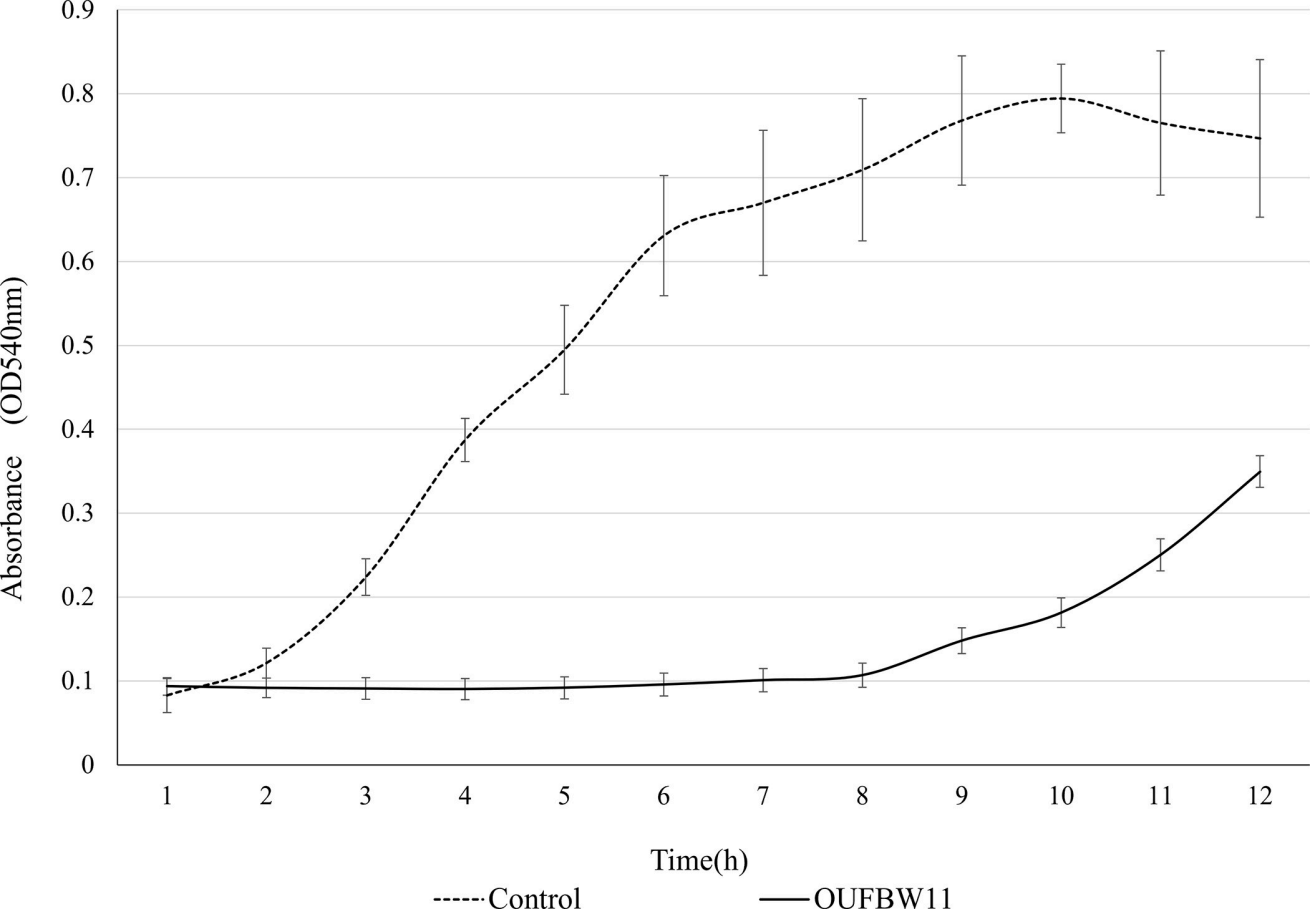
Alamar Blue assay of *C*. *albicans* after exposure to OUFBW. *Candida* metabolic activity was determined by incubating specimens in a TSBD medium containing Alamar Blue and measuring absorbance. (*: *p* < 0.05).

Fig 5 demonstrated that the activity in the control group rapidly increased between 2 to 6 h of incubation and seemingly reached a plateau 7 h after incubation. However, activity in the OUFBW11 group remained low until 9 h of incubation, and activity at 12 h of incubation was still lower than that in the control group.

**Fig 6:**
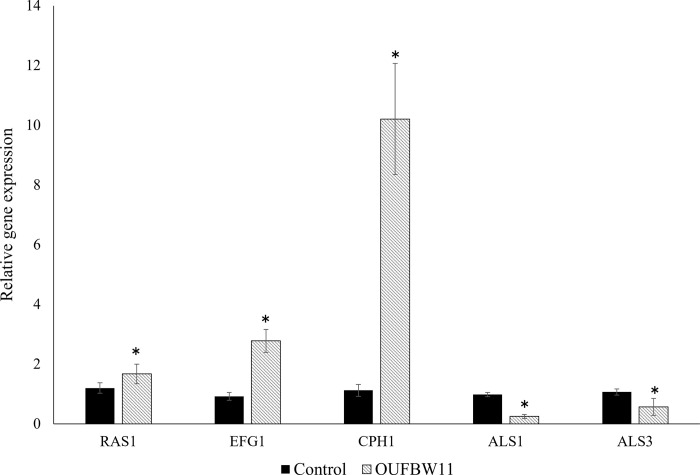
qRT-PCR analysis of the expressions of biofilm formation and hyphal growth. The experiments were repeated three times. (*: *p* < 0.05).

The gene expressions of *C*. *albicans* related to biofilm formation and hyphal growth were evaluated to elucidate the effect of OUFBW (Fig 6). Expressions of biofilm formation-related genes (*ALS1*, *ALS3*) were significantly down-regulated; on the other hand, expressions of hyphal-related genes (*RAS1*, *EFG1*, *CPH1*) were significantly up-regulated.

Table 2 shows the average ± standard deviation Ra and VH values of the sample immersed in OUFBW solutions. In all groups, Ra and VH ranged from 0.1567–0.1584 μm and 19.25–19.74 Hv, respectively. There were no significant differences in either Ra or VH between groups.

**Table 2:** Average ± standard deviation values of surface roughness and Vickers hardness.

	Control	OUFBW6	OUFBW9	OUFBW11	OFEAS
**Ra (μm)**	0.1567 ± 0.006	0.1577 ± 0.006	0.1571 ± 0.009	0.1584 ± 0.009	0.1568 ± 0.007
**VH (Hv)**	19.49 ± 0.876	19.74 ± 0.664	19.25 ± 0.857	19.35 ± 0.937	19.58 ± 1.042

There were no significant differences among the groups in each analysis (Ra: n = 5, VH: n = 5, *p* > 0.05). OFEAS refers to ozone-free electrolyte aqueous solution of OUFBW11. OUFBW, ozone ultrafine bubble water; Ra, surface roughness; VH, Vickers hardness.

## Discussion

The biofilms recovered from discs treated with OUFBW11 and OUFBW9 showed significantly reduced viable counts of *C*. *albicans* compared with the biofilms in the control group. However, the biofilms formed within 24-hour were not removed completely. As shown in the results of the *Candida* growth and Alamar Blue assay, this reducing effect might be due to growth inhibitory of *C*. *albicans*. The results of the *Candida* growth assay showed the effect of OUFBW on the growth rate at 0, 3, and 5 h of incubation, and biofilm growth was suppressed in a concentration-dependent manner. This result was supported by SEM observation. Moreover, the control values of the Alamar Blue absorbance increased 8.75-times fold at 8 h of incubation, whereas OUFBW values increased 1.17-fold at 8 h of incubation, showing almost no changes between pre- and post-incubation values. These results indicate that OUFBW might inhibit biofilm formation by suppressing the growth of *C*. *albicans* that initially adhered to the denture surface.

The mechanisms underlying the inhibitory effect of OUFBW on *C*. *albicans* growth may be explained by previous reports. Ozone is generally known as a powerful oxidant [28]; it is believed that ozone promotes the oxidation of lipids and proteins in the cell wall and cell membranes, and may deactivate fungi by altering its permeability [29]. However, the results of fluorescence microscopy revealed that most of the *C*. *albicans* cells remain alive, even when exposed to high concentration of OUFBW11. PI containing in the LIVE/DEAD kit penetrates through *Candida* cells by damaged cell walls/membranes and emits red fluorescence when PI is bound to DNA [30], suggesting that *Candida* cell walls and membranes were not damaged by OUFBW. On the other hand, it has been reported that periodontal pathogens are sterilized by OUFBW [16]. In addition, a previous study demonstrated that oxidation by ozone is more likely to affect gram-negative anaerobic bacteria *Porphyromonas endodontalis* and *Porphyromonas gingivalis* than fungi [31], and one study found that microorganisms may exhibit bacteriostatic activity when treated with ozonated water in early stages [32]. This difference between *C*. *albicans* and these pathogens may be related to the fact that *Candida*, unlike bacteria, is a fungus and has a cell wall. The fungal cell wall plays an important role in protection against environmental stresses, such as exposure to various agents [33]. Although the reason why OUFBW showed fungistatic effect on *C*. *albicans* cannot be explained within the design of this study, the existence of the cell walls might be one of the factors. In addition, the degree of cell denaturation by ozone oxidation has been reported to be dependent on the amount of ozone, operating time, proteins exposed to ozone, and environmental conditions [34]. Thus, we suggest that the OUFBW conditions used in this study might not be enough to damage the cell walls and membranes of *C*. *albicans* but had a fungistatic effect on *C*. *albicans*.

The result of the real-time PCR supports the reason why OUFBW inhibits the growth of *C*. *albicans*. OUFBW suppressed *ALS1* and *ALS3* gene expression in *C*. *albicans*. The ALS family consists of at least eight members which encode glycoproteins in the cell wall [26, 35]. Of these members, ALS1 and ALS3 have been shown to exhibit adhesive activity and are essential during the adhesion stage of *C*. *albicans* [36]. ALS1 helps to enhance adhesion to endothelial cells and encode proteins producing reproductive tracts that grow in host tissues [37]. ALS1 also has the ability to co-aggregate with bacteria and other fungi, which are essential to pathogenesis and infection [38, 39]. ALS3 is thought to encode a multifunctional protein involved in host cell attachment, biofilm formation, host cell infiltration, and iron acquisition [40–42]. As just described, various studies have reported that the expression of these genes is required to form biofilms, suggesting that OUFBW reduces not only *C*. *albicans* adhesion to dentures but also the biofilm formation by suppressing *ALS1* and *ALS3*.

The yeast-to-hyphal switch is one of important factors of forming *Candida* biofilm [43]. The genes involved in morphogenetic conversion to morphogenic hyphae (i.e., *EFG1*, *CPH1*, and *RAS1*) were upregulated, suggesting that the growth of *Candida* biofilm could be promoted. However, our data overall showed the inhibitory effect on *C*. *albicans* growth. Although we cannot completely explain why the upregulation of these genes is caused after immersing in OUFBW in this study, there is the possibility that the response to environmental stress in *C*. *albicans* is associated with these reactions. It has been reported that the transformation to hyphae of *C*. *albicans* is caused to protect the *Candida* cell from various extracellular stresses, such as elevated pH, hypoxia, and high CO_2_ and GlcNAc levels [44]. In addition, *EFG1* is a central regulator in the formation of *C*. *albicans* biofilms and upregulated along with *ALS3* during hyphal development [45, 46], which suggest that these genes involved in morphogenetic conversion and adhesive activity might make a complex network and be associated with biofilms formation. Therefore, the down regulation of ALS1 and ALS3 might have a potent influence on the inhibition of *C*. *albicans* growth in this study. Further research is needed on the mechanism underlying the influences of OUFBW on genes involved in hyphal growth and adhesion.

Ozone does not damage skin cells unlike other powerful disinfectants [16, 47]. Moreover, since it decomposes into oxygen, it does not leave harmful residues after use. Therefore, ozone can be safely used in humans, and research has identified its potential applications in dentistry [27]. However, half-life of dissolved ozone is about 20 minutes [48]. Furthermore, sufficient care must be taken during the usage of an ozone generator because gaseous ozone is usually used. On the other hand, OUFBW is superior to common ozonated water because we need not to frequently use an ozone generator in that it can be stored for an extended period of time by creating an ion cloud around the bubble. Additionally, OUFBW can be stored in polyethylene terephthalate (PET) plastic bottles and is suitable for home use [49].

The ozone concentrations of OUFBW used in this study were 6 ppm, 9 ppm, and 11 ppm. Previous studies on ozonated water suggest that approximately 2 ppm can kill microorganisms in vitro [50, 51]. In contrast, the bactericidal activity of ozone may decrease in the presence of proteins present in saliva and bacterial biofilms [16]. Another study has shown that approximately 10 ppm of ozone water can effectively reduce bacterial viability even with proteins [52]. A study using ozone water for *C*. *albicans* has also shown that *C*. *albicans* decreased to 10% after soaking in about 10 ppm ozone water [53]. Therefore, we prepared the concentration to 6 ppm, 9 ppm, and 11 ppm assuming that the actual environment in the oral cavity is complex.

Conventionally, ozone decomposes polymers [54], suggesting that using OUFBW may inhibit biofilm formation without damaging the surface of PMMA. Further verification is needed to determine whether OUFBW can be used after denture cleansers as an auxiliary storage solution for *C*. *albicans* growth and biofilm inhibition.

## Conclusion

In conclusion, we demonstrated that OUFBW containing 11 ppm of ozone significantly suppressed the growth and biofilm formation of *C*. *albicans* on the denture base resin. In addition, immersion in OUFBW for 7 days did not cause the deterioration of denture base resin. Our data also suggested that OUFBW has an inhibitory effect on the metabolic function of *C*. *albicans*. Further investigations are required to elucidate details of the mechanism underlying the fungistatic effect of OUFBW on *C*. *albicans* and to analyze the clinical effect of OUFBW on denture wearers.

## Supporting information

S1 File Base data of 24-h Biofilm quantification. (XLSX)

S2 File Base date of time point CFU assay. (XLSX)

S3 File Base date of alamar blue assay. (XLSX)

S4 File Base date of qRT-PCR analysis. (XLSX)

## References

[1] Singh A, Verma R, Murari A, Agrawal A. Oral candidiasis: An overview. J Oral Maxillofac Pathol. 2014;18: 81–85. doi: 10.4103/0973-029X.141325 25364186 PMC4211245

[2] Gleiznys A, Zdanavičienė E, Žilinskas J. Candida albicans importance to denture wearers. A literature review. Stomatologija. 2015;17: 54–66. 26879270

[3] Budtz-Jørgensen E, Mojon P, Rentsch A, Deslauriers N. Effects of an oral health program on the occurrence of oral candidosis in a long-term care facility. Commun Dent Oral Epidemiol. 2000;28: 141–149. doi: 10.1034/j.1600-0528.2000.028002141.x 10730723

[4] Pereira-Cenci T, Del Bel Cury AA, Crielaard W, Ten Cate JM. Development of Candida-as- sociated denture stomatitis. new insights. J Appl Oral Sci. 2008;16: 86–94. doi: 10.1590/s1678-77572008000200002 19089197 PMC4327625

[5] Webb BC, Thomas CJ, Willcox MDP, Harty DW, Knox KW. Candida-associated denture stomatitis. Aetiology and management: a review. Part 1. Factors influencing distribution of Candida species in the oral cavity. Aust Dent J. 1998; 43: 45–50. doi: 10.1111/j.1834-7819.1998.tb00152.x 9583226

[6] Gulati M, Nobile CJ. Biology C. Candida albicans biofilms: development, regulation, and molecular mechanisms. Microbes Infect. 2017;18: 310–321. doi: 10.1016/j.micinf.2016.01.002.Candida PMC4860025 26806384

[7] Enfert C. Hidden killers: persistence of opportunistic fungal pathogens in the human host. Curr Opin Microbiol. 2009;12: 358–364. doi: 10.1016/j.mib.2009.05.008 19541532

[8] Williams DW, Kuriyama T, Silva S, Malic S, Lewis MA. Candida biofilms and oral candidosis: Treatment and prevention. Periodontol 2000. 2011;55: 250–265. doi: 10.1111/j.1600-0757.2009.00338.x 21134239

[9] Mishra NN, Prasad T, Sharma N, Payasi A, Prasad R, Gupta DK, et al. Pathogenicity and drug resistance in Candida albicans and other yeast species. Acta Microbiol Immunol Hung. 2007;54: 201–235. doi: 10.1556/AMicr.54.2007.3.1 17896473

[10] Morschhäuser J. Regulation of multidrug resistance in pathogenic fungi. Fungal Genet Biol. 2010;47: 94–106. doi: 10.1016/j.fgb.2009.08.002 19665571

[11] Yadav V, Garg S, Garg R, Mittal S, Yadav R. Effectiveness of different denture cleansing methods on removal of biofilms formed in vivo. J Cranio Max Dis. 2013;2: 22. doi: 10.4103/2278-9588.113582

[12] Gad MM, Fouda SM. Current perspectives and the future of Candida albicans-associated denture stomatitis treatment. Dent Med Probl. 2020; Volume 57: 95–102. doi: 10.17219/dmp/112861 32307934

[13] Arita M, Nagayoshi M, Fukuizumi T, Okinaga T, Masumi S, Morikawa M, et al. Microbicidal efficacy of ozonated water against Candida albicans adhering to acrylic denture plates. Oral Microbiol Immunol. 2005;20: 206–210. doi: 10.1111/j.1399-302X.2005.00213.x 15943763

[14] Takahashi M, Chiba K, Li P. Free-radical generation from collapsing microbubbles in the absence of a dynamic stimulus. J Phys Chem B. 2007;111: 1343–1347. doi: 10.1021/jp0669254 17253740

[15] Hayashi K, Onda T, Honda H, Ozawa N, Ohata H, Takano N, et al. Effects of ozone nano-bubble water on mucositis induced by cancer chemotherapy. Biochem Biophys Rep. 2019;20: 100697. doi: 10.1016/j.bbrep.2019.100697 31692631 PMC6806368

[16] Hayakumo S, Arakawa S, Takahashi M, Kondo K, Mano Y, Izumi Y. Effects of ozone nano-bubble water on periodontopathic bacteria and oral cells—in vitro studies. Sci Technol Adv Mater. 2014;15: 6996. doi: 10.1088/1468-6996/15/5/055003 27877715 PMC5099676

[17] Leewananthawet A, Arakawa S, Okano T, Daitoku R, Ashida H, Izumi Y, et al. Ozone ultra fi ne bubble water induces the cellular signaling involved in oxidative stress responses in human periodontal ligament fibroblasts. Sci Technol Adv Mater. 2019;20: 589–598. doi: 10.1080/14686996.2019.1614980 31258824 PMC6586087

[18] Zargaran M, Fatahinia M, Zarei Mahmoudabadi A. The efficacy of gaseous ozone against different forms of Candida albicans. Curr Med Mycol. 2017;3: 26–32. doi: 10.18869/acadpub.cmm.3.2.26 29354778 PMC5763895

[19] Takahashi M, Shirai Y, Sugawa S. Free-radical generation from bulk nanobubbles in aqueous electrolyte solutions: ESR spin-trap observation of microbubble- treated water. Langmuir. 2021;37: 5005–5011. doi: 10.1021/acs.langmuir.1c00469 33857377

[20] Tsutsumi-Arai C, Arai Y, Terada-Ito C, Takebe Y, Ide S, Umeki H, et al. Effectiveness of 405-nm blue LED light for degradation of Candida biofilms formed on PMMA denture base resin. Lasers Med Sci. 2019 Sep;34(7):1457–1464. doi: 10.1007/s10103-019-02751-2 30798389

[21] Tsutsumi-Arai C, Takakusaki K, Arai Y, Terada-Ito C, Takebe Y, Imamura T, et al. Grapefruit seed extract effectively inhibits the Candida albicans biofilms development on polymethyl methacrylate denture-base resin. PLoS One. 2019 May 28;14(5):e0217496. doi: 10.1371/journal.pone.0217496 31136636 PMC6538181

[22] Tsutsumi C, Takakuda K, Wakabayashi N. Reduction of Candida biofilm adhesion by incorporation of prereacted glass ionomer filler in denture base resin. J Dent. 2016;44: 37–43. doi: 10.1016/j.jdent.2015.11.010 26655872

[23] Hirasawa M, Tsutsumi-Arai C, Takakusaki K, Oya T, Fueki K, Wakabayashi N. Superhydrophilic co-polymer coatings on denture surfaces reduce Candida albicans adhesion—an in vitro study. Arch Oral Biol. 2018;87: 143–150. doi: 10.1016/j.archoralbio.2017.12.024 29291436

[24] Terada C, Imamura T, Ohshima T, Maeda N, Tatehara S, Tokuyama-Toda R, et al. The effect of irradiation with a 405nm blue-violet laser on the bacterial adhesion on the osteosynthetic biomaterials. Hindawi Int J Photoenergy. 2018;2018.

[25] Tsutsumi-Arai C, Arai Y, Terada-Ito C, Imamura T, Tatehara S, Ide S, et al. Microbicidal effect of 405-nm blue LED light on Candida albicans and Streptococcus mutans dual-species biofilms on denture base resin. Lasers Med Sci. 2021 Apr 30. doi: 10.1007/s10103-021-03323-z 33931832

[26] Hoyer LL, Als T. The ALS gene family of Candida albicans. Trends Microbiol. 2001;9: 176–180. doi: 10.1016/s0966-842x(01)01984-9 11286882

[27] Kumamoto CA, Vinces MD. Contributions of hyphae and hypha-co-regulated genes to Candida albicans virulence. Cell Microbiol. 2005 Nov;7(11):1546–54. doi: 10.1111/j.1462-5822.2005.00616.x 16207242

[28] Tiwari S, Avinash A, Katiyar S, Aarthi Iyer A, Jain S. Dental applications of ozone therapy: a review of literature. Saudi J Dent Res. 2017;8: 105–111. doi: 10.1016/j.sjdr.2016.06.005

[29] Elvis AM, Ekta JS. Ozone therapy: a clinical review. J Nat Sci Biol Med. 2011;2: 66–70. doi: 10.4103/0976-9668.82319 22470237 PMC3312702

[30] Kwolek-Mirek M, Zadrag-Tecza R. Comparison of methods used for assessing the viability and vitality of yeast cells. FEMS Yeast Res. 2014;14: 1068–1079. doi: 10.1111/1567-1364.12202 25154541

[31] Nagayoshi M, Fukuizumi T, Kitamura C, Yano J, Terashita M, Nishihara T. Efficacy of ozone on survival and permeability of oral microorganisms. Oral Microbiol Immunol. 2004;19: 240–246. doi: 10.1111/j.1399-302X.2004.00146.x 15209994

[32] Razak FA, Musa MY, Abusin HAM, Salleh NM. Oxidizing effect of ozonated-water on microbial balance in the oral ecosystem. J Coll Physicians Surg Pak. 2019;29: 387–389. doi: 10.29271/jcpsp.2019.04.387 30925969

[33] Gao Q, Liou LC, Ren Q, Bao X, Zhang Z. Salt stress causes cell wall damage in yeast cells lacking mitochondrial DNA. Microb Cell. 2014 Mar 3;1(3):94–99. doi: 10.15698/mic2014.01.131 28357227 PMC5349227

[34] Karamah EF, Ilmiyah AP, Ismaningtyas N. The application of ozonated water to maintain the quality of tuna meat: the effect of contact time, contact temperature and ozone dosage. 2019;509: 12004. doi: 10.1556/650.2019.31393 31055963

[35] Mourer T, El Ghalid M, d’Enfert C, Bachellier-Bassi S. Involvement of amyloid proteins in the formation of biofilms in the pathogenic yeast Candida albicans. Res Microbiol. 2021;172: 103813. doi: 10.1016/j.resmic.2021.103813 33515679

[36] Otoo HN, Lee KG, Qiu W, Lipke PN. Candida albicans Als adhesins have conserved amyloid-forming sequences. Eukaryot Cell. 2008;7: 776–782. doi: 10.1128/EC.00309-07 18083824 PMC2394976

[37] Fu Y, Rieg G, Fonzi WA, Belanger PH, Edwards JE, Filler SG. Expression of the Candida albicans gene ALS1 in Saccharomyces cerevisiae induces adherence to endothelial and epithelial cells. Infect Immun. 1998;66: 1783–1786. doi: 10.1128/IAI.66.4.1783-1786.1998 9529114 PMC108121

[38] Klotz SA, Gaur NK, De Armond R, Sheppard D, Khardori N, Edwards JE, et al. Candida albicans Als proteins mediate aggregation with bacteria and yeasts. Med Mycol. 2007;45: 363–370. doi: 10.1080/13693780701299333 17510860

[39] Tati S, Davidow P, McCall A, Hwang-Wong E, Rojas IG, Cormack B, et al. Candida glabrata binding to Candida albicans hyphae enables its development in oropharyngeal candidiasis. PLOS Pathog. 2016;12: e1005522. doi: 10.1371/journal.ppat.1005522 27029023 PMC4814137

[40] Almeida RS, Brunke S, Albrecht A, Thewes S, Laue M, Edwards JE, et al. The hyphal-associated adhesin and invasin Als3 of Candida albicans mediates iron acquisition from host ferritin. PLOS Pathog. 2008;4: e1000217. doi: 10.1371/journal.ppat.1000217 19023418 PMC2581891

[41] Liu Y, Filler SG. Candida albicans Als3, a multifunctional adhesin and invasin. Eukaryot Cell. 2011;10: 168–173. doi: 10.1128/EC.00279-10 21115738 PMC3067396

[42] Xiaomin Zhao, Daniels Karla J Oh Soon-Hwan, Green Clayton B, Yeater Kathleen M, Soll David R. LLH. Candida albicans Als3p is required for wild-type biofilm formation on silicone elastomer surfaces. Physiol Behav. 2019;176: 139–148. doi: 10.1099/mic.0.28959-0.Candida PMC2583121 16849795

[43] Nobile CJ, Schneider HA, Nett JE, Sheppard DC, Filler SG, Andes DR, et al. Complementary adhesin function in C. albicans biofilm formation. Curr Biol. 2008 Jul 22;18(14):1017–24. doi: 10.1016/j.cub.2008.06.034 18635358 PMC2504253

[44] Desai JV. Candida albicans hyphae: From growth initiation to invasion. J Fungi (Basel). 2018;4. doi: 10.3390/jof4010010 29371503 PMC5872313

[45] Theberge S, Semlali A, Alamri A, Leung KP, Rouabhia M. C. albicans growth, transition, biofilm formation, and gene expression modulation by antimicrobial decapeptide KSL-W. BMC Microbiol. 2013;13: 246. doi: 10.1186/1471-2180-13-246 24195531 PMC4229313

[46] Argimón S, Wishart JA, Leng R, Macaskill S, Mavor A, Alexandris T, et al. Developmental regulation of an adhesin gene during cellular morphogenesis in the fungal pathogen Candida albicans. Eukaryot Cell. 2007;6: 682–692. doi: 10.1128/EC.00340-06 17277173 PMC1865654

[47] Filippi A. The influence of ozonised water on the epithelial wound healing process in the oral cavity. Clin Oral Surgery, Radiol Oral Med Univ Basel, Switzerland. 2001. Available from: www.oxyplus.net

[48] Kazuo A, Takuro M, Kinya K, Kohei K, Takeshi T, et al. Anti-inflammatory effects of ozonated water in an experimental mouse model. Biomed Rep. 2014;2(5):671–674. doi: 10.3892/br.2014.290 25054009 PMC4106612

[49] Mineaki S, Tatsuya I, Hiroshi T, Masayuki N. Microbicidal Effects of Stored Aqueous Ozone Solution Generated by Nano-bubble Technology. In Vivo. 2017;31:579–583. doi: 10.21873/invivo.11097 28652423 PMC5566906

[50] Kim J-G, Yousef AE. Inactivation kinetics of foodborne spoilage and pathogenic bacteria by ozone. J Food Sci. 2000;65: 521–528. doi: 10.1111/j.1365-2621.2000.tb16040.x

[51] Khadre MA, Yousef AE, Kim J-G. Microbiological aspects of ozone applications in food: A review. J Food Sci. 2001;66: 1242–1252. doi: 10.1111/j.1365-2621.2001.tb15196.x

[52] Hirai K, Ando N, Komada H, Sounai A, Murakami M, Nakayama H. Investigation of the effective concentration of ozonated water for disinfection in the presence of protein contaminants. Biocontrol Sci. 2019;24: 155–160. doi: 10.4265/bio.24.155 31527346

[53] Murakami H, Sakuma S, Nakamura K, Ito Y, Hattori M, Asai A, et al. Disinfection of removable dentures using ozone. Dent Mater J. 1996 Dec;15(2):220–5. doi: 10.4012/dmj.15.220 . 9550021

[54] Lee R, Michelle L. Coote. Mechanistic insights into ozone-initiated oxidative degradation of saturated hydrocarbons and polymers. Physical Chemistry Chemical Physics 2016;18: 24663–24671. doi: 10.1039/c6cp05064f 27545312

